# Supplementary material for: Identification of photoperiod-induced specific miRNAs in the adrenal glands of Sunite sheep (Ovis aries)
Source: Front Vet Sci. 2022 Jul 22;9:888207. doi: 10.3389/fvets.2022.888207 (PMC9354845; doi:10.3389/fvets.2022.888207)
Supplement: Supplementary file 1 [file Data_Sheet_1.ZIP › Supplementary file/miRDeep2 file.zip!/miRDeep2 file/Novel_Structure/SPLP42A2/Novel_122.pdf]

Provisional ID	: NC_019478.2_36328
Score total	: 20.7
Score for star read(s)	: 3.9
Score for read counts	: 12.9
Score for mfe	: 2.3
Score for randfold	: 1.6
Score for cons. seed	:
Total read count	: 37
Mature read count	: 12
Loop read count	: 0
Star read count	: 25

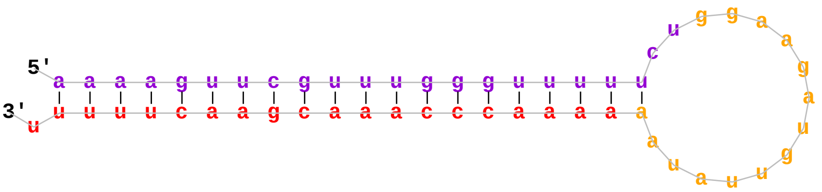

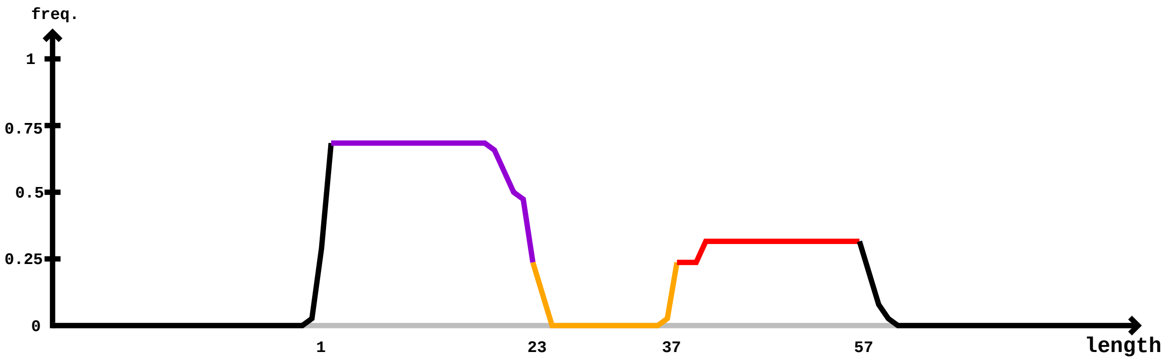


```
                                Star                                   Mature
5'- ugucagauguuuauauuggauuggcuaaaaaguucguuuggguuuuucuggaagauguuauaaaaaacccaaacgaacuuuuuggccaucccaauaaaauaucugcuaggu   -3'    obs
    ugucagauguuuauauuggauuggcuaaaaaguucguuuggguuuuucuggaagauguuauaaaaaacccaaacgaacuuuuuggccaucccaauaaaauaucugcuaggu          exp
    .(.(((((((((.((((((..((((((((((((((((((((((((((...............))))))))))))))))))))))))))..)))))))))))))))).....   reads    mm    sample
    .........................Caaaaaguucguuuggguuuuuc...............................................................   1        1     seq
    ..........................aaaaaguucguuuggguuC..................................................................   2        1     seq
    ..........................aaaaaguucguuuggguuuC.................................................................   2        1     seq
    ..........................aaaaGguucguuuggguuuuuc...............................................................   1        1     seq
    ..........................aaaaaguucguuuggguuuuuA...............................................................   3        1     seq
    ..........................aaaaaguuUguuuggguuuuucu..............................................................   2        1     seq
    ...........................aaaaguucguuuggguu...................................................................   1        0     seq
    ...........................aaaaguucguuuggguuu..................................................................   1        0     seq
    ...........................aaaaguucguuuggguuuG.................................................................   1        1     seq
    ...........................aaaGguucguuuggguuuuu................................................................   1        1     seq
    ...........................aaaaguucguuuggguuuuuA...............................................................   2        1     seq
    ...........................aaaaguucgCuuggguuuuuc...............................................................   2        1     seq
    ...........................aaaaguucguuuggguuuuucG..............................................................   3        1     seq
    ...........................aaaaguuGguuuggguuuuucu..............................................................   1        1     seq
    ...........................aaaaguucguuuggguuuuuGu..............................................................   2        1     seq
    ...........................aaaaguucAuuuggguuuuucug.............................................................   1        1     seq
    ..............................................................aaaaacccaaacgaacuuuuugU..........................   1        1     seq
    ...............................................................aaaacccaaacgaacuuuuu............................   2        0     seq
    ...............................................................aaaaUccaaacgaacuuuuu............................   4        1     seq
    ...............................................................aaaacccaaaGgaacuuuuug...........................   1        1     seq
    ...............................................................aaaaUccaaacgaacuuuuug...........................   1        1     seq
    ..................................................................acccaaacgaacuuuuu............................   1        0     seq
    ..................................................................acccaaacgaacuuuuugU..........................   1        1     seq
    ..................................................................acccaaacgaacuuuCuggc.........................   1        1     seq
```
